# Retinitis pigmentosa and sensorineural deafness associated with a *de novo DHX16* mutation: case report

**DOI:** 10.3389/fgene.2025.1685906

**Published:** 2026-01-05

**Authors:** Lei Wang, Jiyong Gao, Meng Sun, Li Fan, Shuhua Wang, Xue Li, Shuangyu Gu, Bingjuan Han

**Affiliations:** 1 Children’s Medical Rehabilitation Center, Jinan Maternal and Child Health Care Hospital, Jinan, China; 2 Neonatal Disease Screening Center, Jinan Maternal and Child Health Care Hospital, Jinan, China

**Keywords:** retinitis pigmentosa, sensorineural deafness, DHX16 gene mutation, genetic testing, pediatric case

## Abstract

**Background:**

Retinitis pigmentosa and sensorineural deafness are two distinct clinical entities that can be caused by a variety of genetic mutations. The *DHX16* gene, which encodes a protein involved in RNA processing, has been implicated in several genetic disorders. Here, we report a unique case of *de novo DHX16* gene mutation presenting with both retinitis pigmentosa and sensorineural deafness.

**Case Presentation:**

We describe the story of two 2-year-old girls who presented with progressive vision loss and hearing impairment. Both of these cases presented with *de novo* heterozygous mutations in the DHX16 gene. The mutation sites were NM_003587 c.2474C>T and NM_003587.5 c.1360C>T. Ophthalmological examination disclosed the classic stigmata of retinitis pigmentosa, while audiologic assessment revealed bilateral sensorineural hearing loss. Genetic testing identified a *de novo* mutation in the *DHX16* gene, which was not present in the patients’ family histories. The patients were managed with supportive care, including hearing aids to improve their quality of life.

**Conclusion:**

These cases highlight the importance of genetic testing in patients with combined retinitis pigmentosa and sensorineural deafness. Early identification of the underlying genetic mutation can facilitate appropriate management and genetic counseling for affected individuals and their families. Further research is needed to explore the pathophysiological mechanisms and potential therapeutic targets for *DHX16*-related disorders.

## Background

Retinitis pigmentosa (RP) is a group of inherited retinal degenerative diseases characterized by progressive photoreceptor and retinal pigment epithelium degeneration, leading to significant vision loss (Bhatti, 2006; Suleman, 2025). Sensorineural deafness is a type of hearing loss that occurs due to damage to the inner ear, auditory nerve, or central auditory pathways (Saeed et al., 2025). Both conditions can be caused by mutations in various genes, and their co-occurrence can complicate diagnosis and management.

The *DHX16* gene encodes a protein involved in RNA processing and has been associated with several genetic disorders (Drackley et al., 2024). Mutations in this gene can lead to a range of phenotypes, including developmental delay, neuromuscular disease, seizures, ocular nerve and/or retinal degeneration, and sensorineural hearing loss (SNHL) (Paine et al., 2019). Here, we present two cases of *de novo DHX16* gene mutation resulting in both RP and sensorineural deafness. Usher syndrome represents the most typical condition characterized by the co-occurrence of RP and SNHL, inherited in an autosomal recessive manner. Based on the severity of hearing impairment and vestibular dysfunction, it is classified into three subtypes (*USH1–3*), among which USH1 and USH2 are the most prevalent (Delmaghani and El-Amraoui, 2022).

Additionally, Alport syndrome—caused by mutations in the *COL4A3/4/5* genes—may occasionally present with both RP and SNHL, though renal abnormalities remain its predominant feature (Kashtan et al., 2018). Wolfram syndrome (DIDMOAD), resulting from *WFS1* mutations, typically manifests with diabetes mellitus, optic atrophy, and SNHL, with some cases exhibiting RP-like retinal degeneration (Barrett et al., 1995). Mitochondrial disorders, such as maternally inherited diabetes and deafness (MIDD), may also demonstrate RP-like fundus changes in conjunction with SNHL (Murphy et al., 2008). Regarding the *DHX16* gene, there is currently no definitive literature reporting its direct association with the co-occurrence of RP and SNHL.

## Case presentation

**FIGURE 1:**
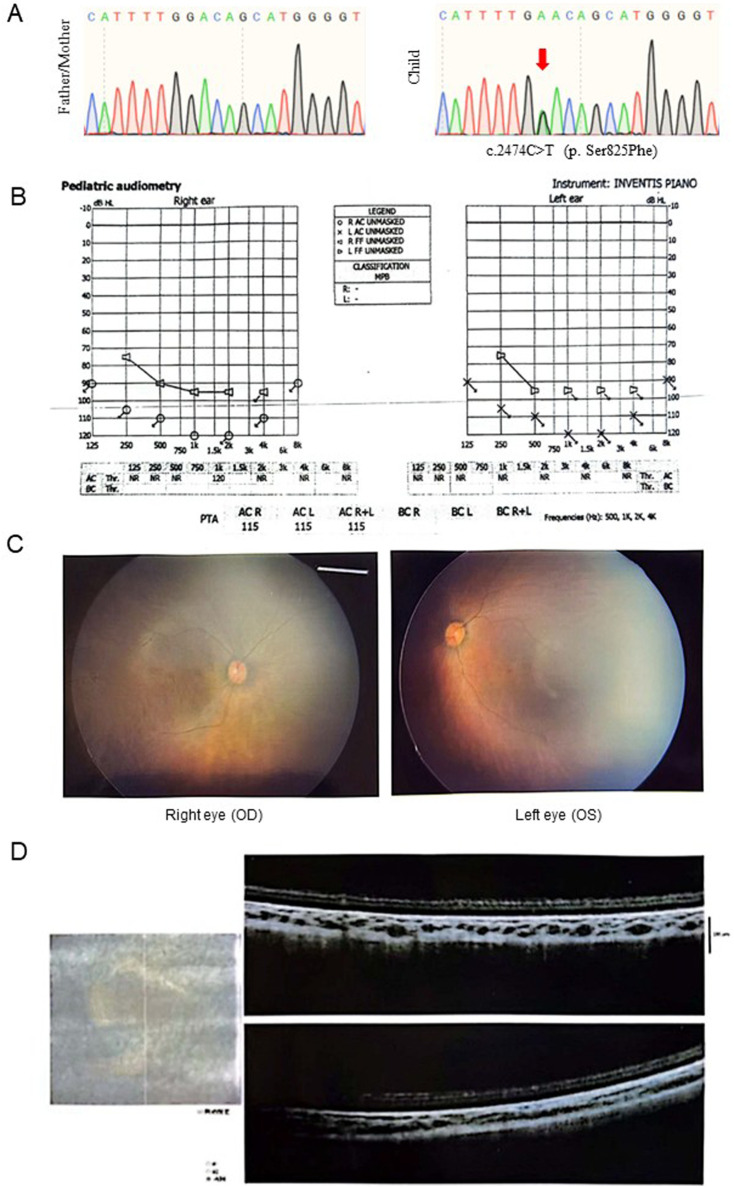
Clinical features of case 1 with Retinitis pigmentosa and sensorineural hearing loss. **(A)** Sanger sequencing results of the genotypes of parents and child. **(B)** Sound pressure level testing used to assess the patient’s hearing level. **(C)** The results of the fundus examination of both eyes. **(D)** The results of the optical coherence tomography (OCT) examination of the patient.

Case 1, a 2-year-old female infant, presented with a complex medical history. Hearing screening performed 3 days after birth revealed failure in one ear. At 1 month of age, the well-child assessment documented absent visual tracking and auditory response. At 3 months of age, follow-up testing indicated bilateral hearing impairment. By 3 months, she also exhibited unstable head control and hypotonia (low muscle tone). Promptly following the hearing diagnosis at 5 months, genetic testing and electromyography (EMG) were initiated due to suspected genetic etiology. Genetic testing identified a *de novo* heterozygous mutation in the *DHX16* gene: NM_003587 c.2474C>T (p. Ser825Phe) (Figure 1A).

Subsequent diagnostic audiological evaluation at 5 months of age confirmed a diagnosis of severe sensorineural hearing loss. Developmental concerns were noted early. Prior to cochlear implantation (CI) at 8 months of age, pre-and post-aided audiometric thresholds were documented as follows: Right ear (unaided): No response across 125–6,000 Hz at 90–120 dB HL. Right ear (aided): Responses observed at 250–2000 Hz with thresholds of 75–95 dB HL. Left ear (unaided): No response across 125–6,000 Hz at 90–120 dB HL. Left ear (aided): Responses detected at 250–500 Hz with thresholds of 75–95 dB HL (Figure 1B).

Ophthalmic evaluation at 1 month of age revealed bilateral lens opacities. By 14 months of age (1 year and 2 months), an ophthalmic examination detected no light perception (NLP), leading to a subsequent diagnosis of Leber congenital amaurosis (LCA). The examination demonstrated findings diagnostic of retinitis pigmentosa, featuring waxy pallor of the optic disks, attenuation of retinal vessels, grayish retinal discoloration, and pigmentary mottling (Figure 1C), with bilateral loss of foveal reflex obscuring anatomical localization of the central fovea (Figure 1D).

**FIGURE 2:**
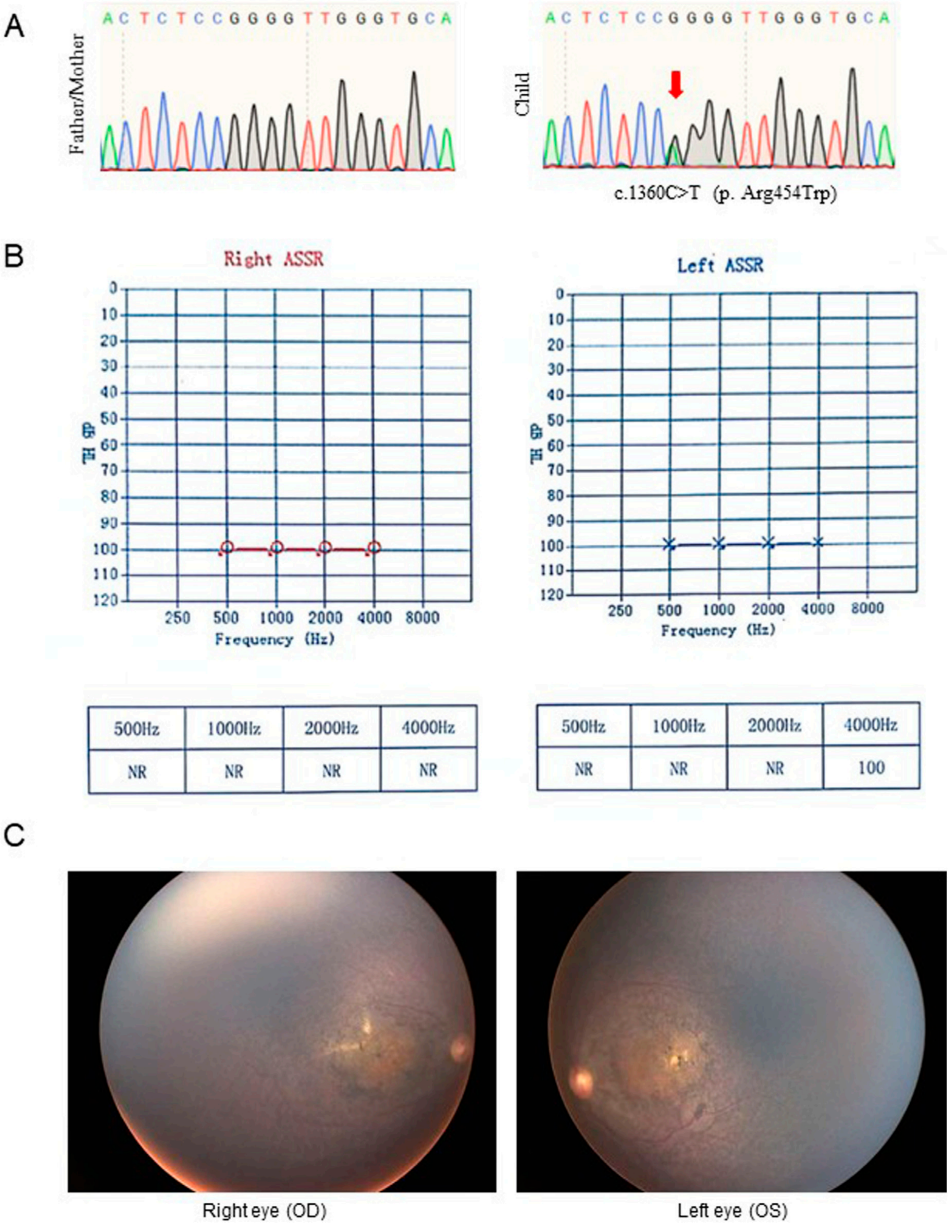
Clinical features of case 2 with Retinitis pigmentosa and sensorineural hearing loss. **(A)** Sanger sequencing results of the genotypes of parents and child. **(B)** The auditory Steady-State Response test of the patient. **(C)** The results of the fundus examination of both eyes.

Case 2, a 2-year-old female infant, presented with a history of failed newborn hearing screening, absent visual tracking, and global developmental delay. Ophthalmologic examination revealed fundus abnormalities. Follow-up audiological assessment at 3 months of age confirmed persistent bilateral hearing impairment. By 7 months of age, a comprehensive diagnostic evaluation established definitive diagnoses of severe sensorineural hearing loss, bilateral retinal degeneration, and macular atrophy. Enrollment in a rare disease research program at 8 months of age included genetic analysis, which identified a *de novo* heterozygous mutation in the *DHX16* gene: NM_003587.5 c.1360C>T (p. Arg454Trp) (Figure 2A). She was ultimately diagnosed with LCA and sensorineural hearing loss.

Auditory steady-state response (ASSR) testing was performed using Eclipse 10 stimuli at modulation rates of 77–100 Hz. Results demonstrated that right ear absence of reproducible responses across 500–4,000 Hz at maximum stimulus intensity of 100 dB nHL. The left ear shows no detectable responses at 500–2000 Hz at 100 dB nHL, but a significant response was present at 4,000 Hz (threshold: 95 dB nHL) (Figure 2B).

At 8 months of age, the infant underwent fundus screening, which revealed retinitis pigmentosa in both eyes with macular lesions. Upon follow-up examination at 2.5 years of age, fundus examination showed diffuse bone-spicule pigmentation in both retinas, involving the macular area. The macula exhibited a yellowish discoloration with pigmentary disturbances (Figure 2C).

The patient was managed with supportive care to improve quality of life. Low-vision aids were provided to assist with daily activities, and hearing aids were fitted to address the hearing impairment. Regular follow-up appointments were scheduled to monitor the progression of the disease and provide ongoing support. Clinical and genetic characteristics of patients with *DHX16* variants are listed in Table 1, and key events and metrics are listed in Table 2.

**TABLE 1:** Clinical and genetic characteristics of patients with *DHX16* variants.

Patient	Variant	Inheritance	Neurologic/neuromuscular	Ocular	Audiologic	Other
Case 1	c.2474C>T	*De novo*	Hypotonia	Retinal degeneration; early-onset retinal degeneration	SNHL	Motor skills delayed
Case 2	c.1360C>T	*De novo*	Delayed motor development	Retinal degeneration (LCA syndrome)	SNHL	Short period of standing

**TABLE 2:** Timeline of patient care: key events and metrics.

Case	Key events	Metrics
Case1 c.2474C>T (p. Ser825Phe)	Absent visual tracking and auditory response (1 month) Bilateral hearing impairment (3 months) Genetic testing (5 months)	Cochlear implantation (CI) (8 months)
Case 2 c.1360C>T (p. Arg454Trp)	Bilateral hearing impairment (3 months) Definitive diagnoses of severe sensorineural hearing loss, bilateral retinal degeneration, and macular atrophy (7 months) Genetic testing (8 months)	Low-vision aids and cochlear implantation (CI) (2.5 years)

**FIGURE 3:**
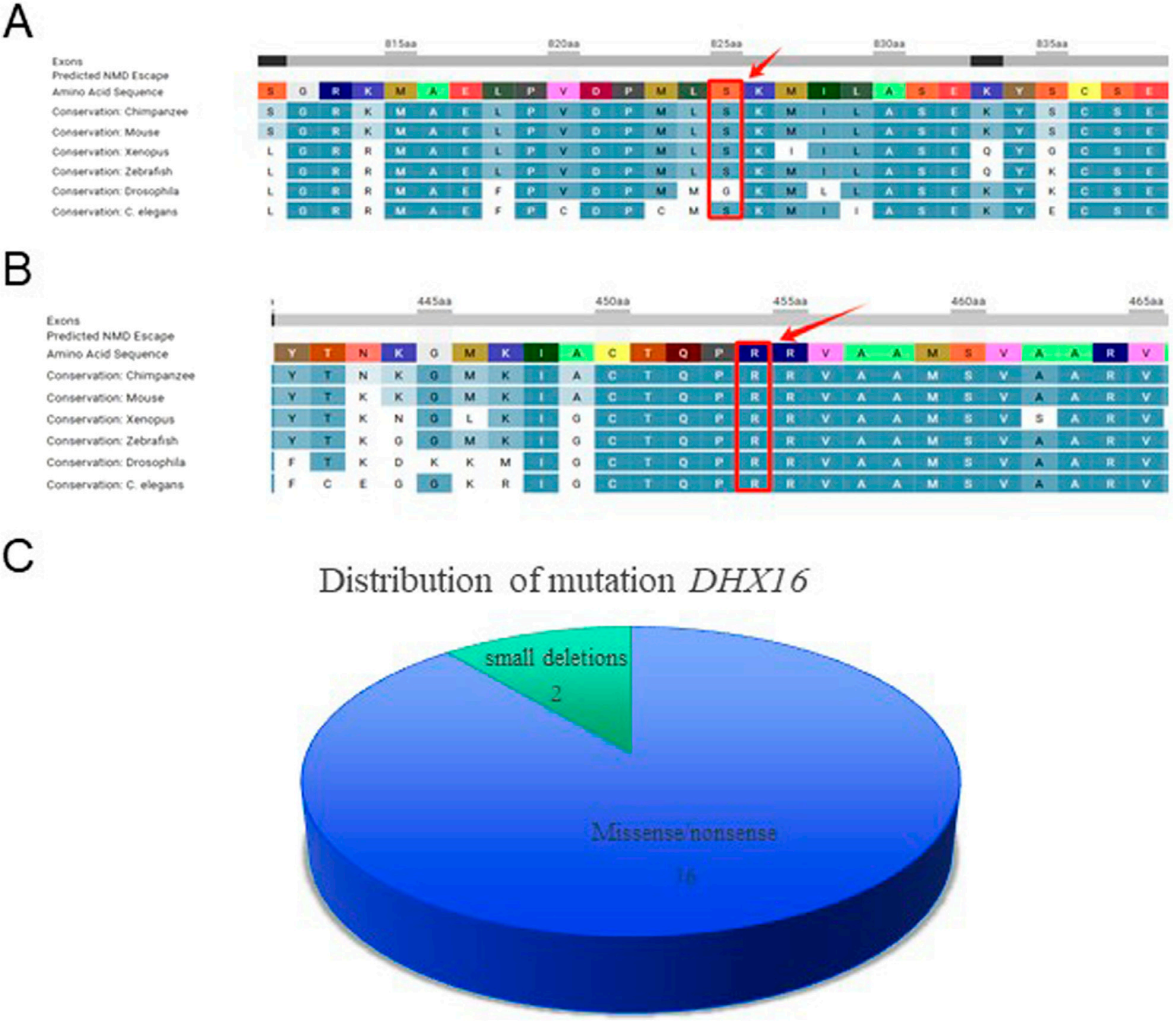
Molecular characterization of pathogenic DHX16 variants: evolutionary conservation analysis and clinical spectrum. **(A)** ClustalX alignment demonstrates evolutionary conservation of the pathogenic DHX16 Ser825Phe variant across eight species. **(B)** ClustalX alignment reveals evolutionary conservation of the Arg454 residue across eight vertebrate and invertebrate species. **(C)** Systematic review identifies 18 pathogenic DHX16 variants (89% missense) in human cases.

ClustalX-based alignment revealed that the amino acid substitution site (Ser825) is evolutionarily conserved across multiple species, including human, chimpanzee, mouse, horse, alpaca, chicken, anole lizard, and *Caenorhabditis elegans* (Figure 3A). *In silico* analysis of the *DHX16* variant c.2474C>T (p. Ser825Phe) further confirmed the high conservation of the Ser825 residue.

Multiple sequence alignment using ClustalX demonstrated that the amino acid substitution site (Arg454) is evolutionarily conserved across diverse species, including human (*Homo sapiens*), chimpanzee (*Pan troglodytes*), mouse (*Mus musculus*), horse (*Equus caballus*), alpaca (*Vicugna pacos*), chicken (*Gallus gallus*), anole lizard (*Anolis carolinensis*), and nematode (*Caenorhabditis elegans*) (Figure 3B). *In silico* analysis of the *DHX16* missense variant c.1360C>T (p. Arg454Trp) further confirmed the high degree of conservation at this residue position.

We systematically reviewed all published *DHX16* variants in human cases, identifying a total of 18 distinct *DHX16* variants (including those reported in the present study). Among these, missense variants predominated (n = 16, 89%), while small deletions accounted for the remaining cases (n = 2, 11%) (Figure 3C). When we checked the frequency of the identified variants in population databases, we found that the variants c.2474C>T and c.1360C>T were absent from major population databases such as 1,000 Genomes (2015aug_all) and ESP6500SI. Based on the ACMG/AMP guidelines, the variant c.2474C>T is classified as likely pathogenic, supported by its confirmed *de novo* origin with absence in both parents, its absence from control populations, and the high concordance between the patient’s specific clinical presentation and a disease with a monogenic etiology associated with this gene. The variant c.1360C>T is classified as pathogenic according to the ACMG/AMP guidelines. This definitive classification is supported by multiple lines of evidence: its established presence in literature databases as a known disease-causing variant, the confirmation of its *de novo* origin through parental segregation studies, its absence in control populations, and the high specificity of the patient’s phenotype to a monogenic disorder associated with this gene. We also checked the pathogenicity of the identified variant on different online software (Table 3).

**TABLE 3:** Pathogenicity of the variant c.2474C>T on different online software.

Variant	Analysis tools and pathogenicity							
c.2474C>T	REVEL	U (0.577)	Polyphen2	Probably_damaging (0.938)	MutationTaster	Disease_causing (1)	MCAP	P (0.05649927)
	SIFT	Damaging (0.001)	LRT	D (0)	GERP	Conserved (4.89)	dbscSNV	-
	SPIDEX	0.0438	SpliceAI	-	ClinPred	0.99559170	AlphaMissense	Pathogenic (0.9883)
c.1360C>T	REVEL	U (0.484)	Polyphen2	Probably_damaging (0.999)	MutationTaster	Disease_causing (1)	MCAP	P (0.13698229)
	SIFT	Damaging (0)	LRT	D (0)	GERP	Conserved (3.43)	dbscSNV	-
	SPIDEX	0.1513	SpliceAI	-	ClinPred	0.99936610	AlphaMissense	Pathogenic (0.9976)

## Discussion and conclusion

This case highlights the importance of genetic testing in patients presenting with combined retinitis pigmentosa and sensorineural deafness. The identification of a *de novo* mutation in the *DHX16* gene underscores the genetic heterogeneity of these conditions and the potential for novel mutations to cause complex phenotypes. Our patients exhibited significant neurological features beyond the core phenotypes of RP and SNHL, which have not been emphasized in previous reports linking *DHX16* to isolated sensory impairments. Specifically, both patients presented with global developmental delay, including hypotonia (Case 1) and delayed motor development (Case 2). These findings are crucial as they suggest that *DHX16*-related disorders may encompass a broader neurodevelopmental spectrum, rather than being confined to purely sensory deficits. This expands the clinical profile associated with *DHX16* mutations and distinguishes our cases from the classic presentation of syndromes like Usher syndrome, where such pronounced global developmental delay is not a hallmark feature. The concomitant presentation of RP and SNHL is a hallmark of Usher syndrome (USH). This genetically heterogeneous condition is classified into three types: the most severe form, *USH1*, caused by mutations in genes that include *MYO7A* and *CDH23* (Nakanishi et al., 2010). The most prevalent form, *USH2*, primarily results from defects in *USH2A* (Zhu et al., 2021). *USH3* is characterized by variable progression and is mainly associated with mutations in the *CLRN1* gene (Wang et al., 2024).

The *DHX16* gene is involved in RNA processing, and its mutations can lead to a variety of clinical manifestations (Drackley et al., 2024). The co-occurrence of retinitis pigmentosa and sensorineural deafness in these two patients suggests a possible role of the *DHX16* gene in both retinal and auditory function. We have prepared a mutation table for the *DHX16* report and mentioned the zygosity of the variations as well as the reported phenotypes for each variation (Table 4).

**TABLE 4:** Mutations of DHX16 and reported phenotypes for each variant.

Variant	Inheritance	Phenotype	References
c.2033A>G (p. Glu678Gly)	*De novo*	Neuromuscular disease, hearing loss, retinal degeneration, and previously unreported phenotypic features, including mitochondrial deficiency and primary ovarian insufficiency	Drackley et al. (2024)
c.2091G>T (p. Gln697His)	*De novo*	Small size; short limbs; dysmorphic facial features; enlarged, cystic kidney	Paine et al. (2019)
c.2021C>T (p. Thr674Met)	*De novo*	Myopathy with isolated necrotic fibers; elevated creatine kinase (CK); abnormal gait; hypertrophic calf muscles; peripheral neuropathy; epilepsy; non-ambulation; tapetoretinal degeneration; total vision loss; flexion contractures	
c.1280G>A (p. Gly427Glu)	*De novo*	Severe congenital hypotonia; denervating motor neuropathy; bilateral talipes equinovarus; poor growth and feeding; respiratory distress; flexion contractures	
c.1744T>A (p. Phe582Ile)	*De novo*	Hypotonia; infantile spasms; chorioretinal lacunae; depigmentation around optic nerve; poor visual tracking; agenesis of corpus callosum; subependymal heterotopia	
c.2474C>T (p. Ser825Phe)	*De novo*	Sensorineural deafness, retinitis pigmentosa, and waxy pallor of the optic discs, attenuation of retinal vessels, grayish retinal discoloration, and pigmentary mottling	This study
c.1360C>T (p. Arg454Trp)	*De novo*	Severe sensorineural hearing loss, bilateral retinal degeneration, and macular atrophy	This study

Mutations in pre-mRNA splicing factors represent the second-most common cause of autosomal dominant retinitis pigmentosa (adRP) and constitute a major source of vision loss (Hafler et al., 2016). For instance, pathogenic variants in the pre-messenger RNA (pre-mRNA) splicing factor 31 (*PRPF31*) gene cause autosomal dominant retinitis pigmentosa. The key genes that have been identified so far include *PRPF31, PRPF3, PRPF4, PRPF6, PRPF8*, and *SNRNP200*, which encode the core components of the spliceosome. *DHX16* may be functionally analogous in this context (Stephenson et al., 2025).

Retinal photoreceptors and cochlear hair cells exhibit a shared vulnerability to degeneration, primarily attributed to their intrinsically high metabolic demands and heightened sensitivity to apoptotic pathway activation (Liu et al., 2007). Mutations in *DHX16* disrupt pre-mRNA splicing fidelity, resulting in aberrant transcripts for key genes essential for retinal photoreceptor and cochlear hair cell function, including those involved in phototransduction and hair cell ciliary structure/function.

*In vitro* studies demonstrate that mutant *DHX16* impairs spliceosome function, resulting in the nuclear accumulation of unspliced pre-mRNA from multiple intron-containing genes. This defect stems from the mutant protein’s formation of a catalytically inactive spliceosomal complex through its interaction with the G-patch protein GPKOW, exerting a dominant-negative effect (Bohnsack et al., 2021; Gencheva et al., 2010). The nuclear accumulation of aberrant transcripts exerts detrimental effects on cellular function through both the toxicity of their high abundance and the concurrent reduction in functional protein expression, as unspliced mRNAs retained within the nucleus are unavailable for translation. Pathogenic variants in other DExD/H-box RNA helicase superfamily members cause comparable phenotypes, often characterized by aberrant central nervous system (CNS) development, which may or may not include extra-CNS anomalies (Paine, Posey, Grochowski, Jhangiani, Rosenheck, Kleyner, Marmorale, Yoon, Wang, Robison, Cappuccio, Pinelli, Magli, Akdemir, et al., 2019; Blok et al., 2015). Future diagnostic screening panels for retinitis pigmentosa with concurrent hearing loss should consider including *DHX16.* Targeted therapeutic interventions for *DHX16*-related pathology remain an unmet clinical need.

Retinitis pigmentosa and other degenerative eye diseases have long been considered “irreversible” blinding disorders, characterized by symptoms such as night blindness and progressive visual field constriction, often leading to gradual vision loss and impaired quality of life. While traditional approaches such as gene therapy, optogenetics, or earlier-generation stem cell treatments have demonstrated limited efficacy, they remain constrained by significant technical challenges. Currently, multiple therapeutic strategies are being explored for patients with RP. Notably, the latest breakthrough involving human ciliary margin-derived retinal stem cells (hNRSCs) has achieved precise localization, functional validation, and retinal repair in animal models for the first time, offering end-stage patients a transformative potential—from “disease delay” to “tissue regeneration” (Liu et al., 2025).

Currently, clinical interventions for SNHL include hearing aids, vasodilators, neurotrophic drug therapy, and cochlear implants. However, these approaches cannot fully restore hearing but only provide partial improvement, with their efficacy remaining limited—particularly for patients with profound deafness (Kelleci and Golebetmaz, 2023). In recent years, alongside advancements in biotechnology, stem cell therapy has emerged as a prominent research focus in otology. Upon damage to the normal ear structure, stem cells can leverage their multipotent differentiation potential to regenerate functionally and morphologically equivalent tissues, migrating to the injured cochlear regions to facilitate repair (Bergman et al., 2021). This mechanism precisely addresses the inherent irreplaceability of inner ear hair cells, offering a promising therapeutic strategy (Mohammadian et al., 2017; Chorath et al., 2020). Currently, inducing functional hair cells via inner ear stem cell differentiation to replace damaged hair cells is considered to be a feasible treatment for sensorineural hearing loss (Qi et al., 2024).

Further research is needed to elucidate the pathophysiological mechanisms underlying *DHX16*-related disorders and to identify potential therapeutic targets.

In conclusion, this case report emphasizes the significance of genetic testing in diagnosing and managing patients with combined retinitis pigmentosa and sensorineural deafness. Early identification of the underlying genetic mutation can facilitate appropriate management strategies and genetic counseling for affected individuals and their families. Continued research into the role of the *DHX16* gene in retinal and auditory function is essential for advancing our understanding and treatment of these disorders. The identification of *de novo DHX16* mutations in these patients with concurrent LCA and SNHL carries significant implications for both clinical practice and research. These cases underscore the necessity of incorporating *DHX16* into targeted genetic screening panels for neonates presenting with failed hearing screening or early-onset visual impairment, particularly when accompanied by developmental delay. The conserved nature of the mutated residues (Ser825 and Arg454) across species and their predicted impact on RNA splicing suggest a shared molecular mechanism underlying both retinal and cochlear pathology, possibly through disrupted processing of photoreceptor- and hair cell-specific transcripts. From a therapeutic standpoint, while current interventions such as cochlear implantation and low-vision aids provide symptomatic relief, emerging RNA-targeted approaches and stem cell therapies offer promising avenues for addressing the root cause of *DHX16*-related dysfunction. Early genetic diagnosis could substantially reduce healthcare costs by avoiding prolonged diagnostic odysseys while enabling timely intervention to preserve residual sensory function. Moving forward, concerted efforts should focus on establishing animal models to validate genotype–phenotype correlations, expanding multicenter studies to characterize variant-specific clinical manifestations, and conducting rigorous cost-benefit analyses of cascade genetic testing for at-risk family members. These cases highlight how rare genetic disorders can serve as models for understanding fundamental biological processes while simultaneously driving innovations in precision medicine for sensory impairments.

## Data Availability

The original contributions presented in the study are included in the article/supplementary material; further inquiries can be directed to the corresponding author.

## Abbreviations

ASSR, Auditory steady-state response; CNS, Central nervous system; EMG, Electromyography; LCA, Leber congenital amaurosis; RP, Retinitis pigmentosa; SNHL, Sensorineural hearing loss.
